# Anatomy teaching with portable ultrasound to medical students

**DOI:** 10.1186/1472-6920-12-99

**Published:** 2012-10-22

**Authors:** Meenakshi Swamy, Roger F Searle

**Affiliations:** 1 School of Medicine and Health, The Holliday Building, Durham University Queen's Campus, University Boulevard, Stockton on Tees, TS17 6BH, UK; 2 Anatomy and Clinical Skills, School of Medical Sciences Education Development, The Medical School, Newcastle University, Newcastle upon Tyne, NE2 4HH, UK

## Abstract

**Background:**

Medical students as future clinicians will apply their anatomy knowledge in medical imaging. There are various radiological resources available for the medical students to learn anatomy and contextualise it to the clinical setting. Ultrasound is a safe and non- invasive imaging procedure commonly used in clinical practice. This study aimed to use portable ultrasound and evaluate its impact as an adjunct to cadaveric anatomy teaching together with cross sectional anatomy images and line diagrams.

**Methods:**

Ultrasound teaching was incorporated into upper limb and lower limb anatomy practical dissecting room sessions. The number of medical students who participated was 121 students from the year 2008 - 2009 and 94 students from the year 2009- 2010. The students were divided into groups of 15-20. Initially ultrasound demonstration was carried out on a volunteer and then the students were given the opportunity to use the ultrasound and identify normal anatomical structures visualized on images. For the students in the year 2009- 2010, ultrasound teaching was supplemented with cross sectional anatomy images and line diagrams. Questionnaires were distributed with seven questions rated using four point Likert scale and free text. Qualitative data was analysed using 2- proportion Z test and Fischer's exact test.

**Results:**

The number of students in the 2009-2010 year group who were confident in interpreting ultrasound images increased significantly when compared to the 2008-2009 year group of students. The majority of students were able to identify structures like bone, muscles and blood vessels on ultrasound images. There was a significant increase in the number of students who found the ultrasound teaching useful and also those who regarded ultrasound to have improved understanding of anatomy considerably.

**Conclusions:**

Ultrasound acts as a useful adjunct to teach anatomy in a clinical context to medical students. The use of cross sectional anatomy images and line diagrams together can aid ultrasound image orientation of structures during these sessions. Early exposure to this imaging technology may prime students for later encounters with ultrasound during clinical practice.

## Background

Anatomy is one of the important cornerstones of medicine. Traditional way of anatomy teaching using cadavers is universally practiced and has been considered as essential to medical learning [1]. In the conventional setup, anatomy was taught as a preclinical component. At present medical students come in contact with patients as early as the first week of medical training in many of the medical colleges in the UK to contextualise learning in accordance to the recommendations of the GMC [2,3]. However, there is an ongoing debate taking place in medical education regarding anatomy teaching methods following the recommendations of the GMC that there should be streamlined anatomy teaching with integration of clinical sciences. These include the study of anatomy of the living body through peer physical examination and/or body painting and the use of medical imaging methods such as X rays and Ultrasound [4,5].

Ultrasound is safe and has the advantage of providing visualisation of structures and their movement in a non invasive manner. It could potentially be an effective method to enhance and facilitate the learning of anatomy in a clinically relevant manner. Ultrasound has evolved rapidly in the last few decades as an imaging modality and comprises almost 25% of imaging worldwide [6]. Recent NICE guidelines encourage use of ultrasound for many clinical procedures including insertion of a central venous line in elective situations and ultrasound guided foam sclerotherapy for varicose veins. Using portable ultrasound is well documented in settings like emergency medicine, anaesthesia and cardiology, and other specialities are now seeking training in using them [5]. This would mean that there is more likelihood of medical students encountering ultrasound images in clinical practice. It can therefore be regarded as necessary and timely to expose medical students to ultrasound technology, training and images.

Currently, ultrasonography as a teaching modality is being explored in anatomy and clinical skills teaching in undergraduate medical education. Previous studies have found ultrasound as a helpful educational resource with high student satisfaction for teaching cardiovascular/ renal anatomy [7] and for demonstrating organs, forearm muscles/ vessels [8]. It is reported that using cadavers and imaging together improves the students' ability to identify anatomical structures and provides long term knowledge retention [9].

Hence the objective of our study was to use portable ultrasound as an adjunct to cadaveric upper and lower limbs anatomy teaching and to explore the best way of using it as a tool in teaching anatomy. We compared ultrasound with and without additional interpretative line diagrams as an initial step, and found that there was no significant difference in their confidence in interpreting ultrasound images between the two groups. We then introduced cross sectional anatomy images, and found a significant increase in the number of students who were confident in identifying particular structures between the year groups.

## Methods

Portable ultrasound was used to show the normal living anatomy of upper and lower limbs as a part of the practical dissecting room sessions during the Thought, Senses and Movement (TSM) module. It was carried out after upper limb and lower limb anatomy teaching on cadavers and was used as an interactive round up session. A total of 121 second year medical students during the year 2008 - 2009 and 94 students the following year, 2009- 2010 were included in the study. The students had no previous ultrasound experience. They were taught by anatomy demonstrators who were doctors and were familiar with the use of portable ultrasound and the interpretation of images. The students were divided into 2 groups in each year. Within each group, they were further subdivided into 15- 20 for the ultrasound demonstration. After a brief introduction to the mechanics of ultrasound and how to use the portable ultrasound machine, the ultrasound demonstration of anatomy structures was performed for 15-20 minutes under good lighting conditions with projection of images on high quality audio visual equipment so that the students could clearly see and appreciate the structures better. The demonstration was carried out on a volunteer who had given written informed consent and had been pre-scanned for any abnormality or features requiring further clinical investigation. The demonstration included muscles, blood vessels with blood flow, tendons with movements, nerves, bones and other structures (e.g., menisci) in the respective regions of the upper/ lower limbs and the continuation of these structures if any in the other regions. The medical students were actively engaged during the ultrasound demonstration as they were constantly questioned on relevant anatomy. The students were then given the opportunity to use the ultrasound and identify structures for about 10- 15mins on each other. Students were informed about the session beforehand and verbal consent was obtained. There were 4 portable ultrasound machines available for the students to use and they were setup in a dedicated bay. For the second group of 72 students in the year 2008- 2009, ultrasound teaching was supplemented with line diagrams drawn on the white board as the students in the first group fed back that it was difficult to identify and differentiate structures while interpreting the two dimensional ultrasound images (Table 1). During 2009- 2010 ultrasound teaching for all the students was supplemented with cross sectional anatomy images along with line diagrams for orientation and to help students correlate ultrasound images and living anatomy (Table 1).

**Table 1:** Outline of the structure of the study

**2008**-**2009**		**2009**-**2010**
First group of students	Second group of students	Ultrasound with cross-sectional anatomy images and line diagrams
Ultrasound	Ultrasound with line diagrams	

Feedback questionnaires with seven questions rated using 4 point Likert scale and free text (Additional file 1: Appendix 1) were distributed at the end of the session both the year groups and qualitative data was analysed using 2-proportion Z test and Fischer's exact test.

Ethical approval was obtained from Faculty of Medical Sciences Ethics Committee, Newcastle University (Reference number - 00537/2012).

## Results

Responses were received from all the medical students in both year groups (Table 2).

**Table 2:** Comparison of positive responses of the medical students in both the year groups

**Number of students**	**2008**-**2009 n=121**	**2009**-**2010 n=94**	**P value**
Ultrasound teaching			
Useful/essential	63%	78%	0.018
Easy/able to identify following structures			
Bone	70%	95%	<0.001
Muscle	52%	83%	<0.001
Blood vessel	54%	75%	0.001
Tendons	32%	52%	0.002
Nerves	13%	23%	0.042
Improved understanding of anatomy			
Quite a lot/ Considerably	22%	40%	0.003
Enjoyable/ Recommend to a colleague	55%	63%	0.2

In the year group 2008- 2009, there was no statistically significant difference seen in students' responses whether or not the line diagram was used.

When the responses of the two year groups of students were compared (2008-2009 vs. 2009-2010), there was a statistically significant increase in the number of students who were confident in interpreting ultrasound images by identifying the anatomical features. Students who found ultrasound teaching useful and those who regarded ultrasound to have improved their understanding of anatomy, also increased significantly in the year 2009-2010 (Table 2). Moreover, although students in the year group 2009-2010 did not differ from the 72 students in the 2008-2009 group in finding the line diagram useful, the number of students who were confident that they were able to translate most/ all structures on the line diagram onto the ultrasound images increased significantly in the year 2009-2010 (Table 3). The students in the year group 2009-2010 had cross-sectional anatomy images as well as line diagrams to aid in interpretation of images.

**Table 3:** **Comparison of students'responses regarding the use of line diagrams**

**Number of students**	**2008**-**2009**	**2009**-**2010**	**P value**
	**n=72**	**n=94**	
Line diagram			
Useful/ essential	55%	57%	0.85
Able to translate most/ all structures	36%	57%	0.01

Some of the free text comments that the students made in the year group 2008- 2009 included “Very helpful in reinforcing anatomy taught using cadavers”, “Session was advantageous”, “More sessions and more exposure”, “Very difficult to understand/ orientate”, “Line diagrams are very useful to orientate”. The year group 2009- 2010 commented “More clinically relevant information”, “They are useful as they will be applicable to our future as doctors”, “Is best used as a roundup session after the limb anatomy has been completed”, “Smaller groups and more time to practice”.

## Discussion

In medical practice, ultrasound technology is being used since late 1940 and early 1950, and has progressed greatly over time. It is predicted that ultrasound equipment will become more compact and will be the new stethoscope of the future [8,10]. It can therefore be argued that it is important to introduce ultrasound and its significance in undergraduate teaching in the early years of their education.

In our study, portable ultrasound was used as an adjunct to cadaveric upper and lower limbs anatomy teaching to reinforce their anatomy knowledge and to ensure that the students become familiar with ultrasound images. We chose upper and lower limb anatomy as most of the anatomical structures can be visualized. The dynamic nature of this investigation and interpretation of ultrasound images can be challenging to the Phase 1 medical students (pre-clinical). Hence this was supplemented with line diagrams and/ cross sectional anatomy images.

For the students of the year group 2009-2010, cross sectional anatomy images with line diagrams were used alongside ultrasound demonstration to help the students orientate and thus identify structures while interpreting the two dimensional ultrasound images of upper and lower limbs. It was found that the confidence of these students to identify structures increased significantly when compared to the 2008-2009 year group who did not have the benefit of cross sectional anatomy images. There was also a statistically significant increase in the number of students who found the session useful and felt that it had improved their understanding of anatomy. The number of students able to translate most/ all of the structures on the line diagram to identify structures on the ultrasound images increased significantly in the 2009-2010 year group.

Studies have shown that medical students value the use of ultrasound, and this is confirmed by our finding. Integrated anatomy, clinical skill and radiological cardiovascular/ renal sessions focussed on small group problem based learning are valued highly by students on evaluation. The students have considered the use of ultrasound as an innovative, exciting and engaging approach to stimulate learning of clinical anatomy with enhancement of their reasoning skills [7]. Although 95.7% of 2^nd^ year medical students regarded ultrasound as a useful tool when used for demonstrating organs and forearm muscles and vessels, their understanding of ultrasound images was inadequate [8]. In this study, 78% of students in the year group 2009-2010 have found ultrasound useful and among the structures identified, most students could identify bone (95%) followed by muscle (83%) and blood vessel (75%). About half of the students could identify tendons (52%) while the least identified structure was nerves (23%).

Limitations of the study were that the study was performed in a single medical school with only two cohorts of students and the study did not measure knowledge per se but rather confidence in knowledge.

## Conclusions

To our knowledge, this study is novel because the impact of ultrasound with cross sectional anatomy images and line diagrams for teaching anatomy of upper and lower limbs has not been reported. It has shown that this can be a useful adjunct in teaching anatomy to medical students. Anatomy teaching using ultrasound in Phase 1 (year 1& year 2) medical curriculum can act as bridging tool to integrate anatomy learning and clinical practice by helping the students to apply anatomical knowledge to interpret normal ultrasound images and to be more prepared when they encounter abnormalities during their clinical years of medicine and as clinicians in their future practice. It can also increase the students' awareness of the importance of anatomy knowledge in clinical practice.

## Competing interests

The authors declare that they have no competing interests.

## Authors' contributions

MS was involved in conception, design, acquisition, analysis, interpretation of data, drafting of manuscript and literature review. RFS was involved in conception, design, revising it critically and final approval of the manuscript. Both authors read and approved the final manuscript.

## Pre-publication history

The pre-publication history for this paper can be accessed here:

http://www.biomedcentral.com/1472-6920/12/99/prepub

## Supplementary Material

Additional file 1 **Appendix 1.** Questionnaire. Click here for file

## References

[B1] McLachlan JC Bligh J Bradley P Searle J Teaching anatomy without cadavers Medical Education 2004 38 418 424 10.1046/j.1365-2923.2004.01795.x 15025643

[B2] General Medical Council Tomorrow's Doctors 1993 GMC, London

[B3] General Medical Council Good Medical Practice 1995 GMC, London

[B4] McLachlan JC Patten D Anatomy teaching: ghosts of the past, present and future Medical Education 2006 40 243 253 10.1111/j.1365-2929.2006.02401.x 16483327

[B5] Patten D Donnelly L Richards S Studying living anatomy: the use of portable ultrasound in the undergraduate medical curriculum International journal of clinical skills 2010 4 72

[B6] Shapiro RS Ko PP Jacobson S A pilot project to study the use of ultrasonography for teaching physical examination to medical students Comput Biol Med 2002 32 403 409 10.1016/S0010-4825(02)00033-1 12356490

[B7] Tshibwabwa ET Groves HM Integration of ultrasound in the education programme in anatomy Medical Education 2005 39 1143 1172 10.1111/j.1365-2929.2005.02355.x 16262819

[B8] Heilo A Hansen AB Holck P Laerum F Ultrasound ‘electronic vivisection’ in the teaching of human anatomy for medical students Eur J Ultrasound 1997 5 203 207 10.1016/S0929-8266(97)00015-3

[B9] Miles KA Diagnostic imaging in undergraduate medical education: an expanding role Clin Radiol 2005 60 7 742 745 10.1016/j.crad.2005.02.011 15978883

[B10] Hoppmann R Michell WE Carter JB McMahon C Lill PH Brownlee NA Carnevale KA Ultrasound in second year pathology medical education Journal of the South Carolina Academy of Science 2008 7 1 11 12

