# Supplementary material for: Anatomy teaching with portable ultrasound to medical students
Source: BMC Med Educ. 2012 Oct 22;12:99. doi: 10.1186/1472-6920-12-99 (PMC3507803; doi:10.1186/1472-6920-12-99)
Supplement: Additional file 1 — Appendix 1. Questionnaire. [file 1472-6920-12-99-S1.doc]

**Appendix 1**

**Questionnaire**

**Ultrasound use in Undergraduate Anatomy Teaching**

**1. Did you find the teaching on Ultrasound images of the limbs useful?**

No use [ ] Limited use [ ] Useful [ ] Essential [ ]

**2. How easily were you able to identify the following structures?**

***a. Bone***

Not able to identify [ ] With difficulty [ ] Able to identify [ ] Easy to identify [ ]

***b. Muscle***

Not able to identify [ ] With difficulty [ ] Able to identify [ ] Easy to identify [ ]

***c. Vessels***

Not able to identify [ ] With difficulty [ ] Able to identify [ ] Easy to identify [ ]

***d. Nerves***

Not able to identify [ ] With difficulty [ ] Able to identify [ ] Easy to identify [ ]

***e. Tendons***

Not able to identify [ ] With difficulty [ ] Able to identify [ ] Easy to identify [ ]

**3. Did you find demonstration of the living anatomy with Ultrasound improved your understanding of anatomy in general?**

No [ ] Somewhat [ ] Quite a lot [ ] Considerably [ ]

**4. Did you find the line diagram (for orientation during demonstration) useful? (Leave this question and the next blank if you were not demonstrated with the help of line diagrams)**

No use [ ] Limited use [ ] Useful [ ] Essential [ ]

**5. Were you able to translate the structures represented on the diagram to those demonstrated with the ultrasound probe?**

None [ ] Some [ ] Most [ ] All [ ]

**6. Would you recommend these sessions to a colleague?**

No [ ] Possibly [ ] Probably [ ] Yes [ ]

**7. Any suggestions or comments**
